# Alexander the Great and West Nile Virus Encephalitis

**DOI:** 10.3201/eid1007.040039

**Published:** 2004-07

**Authors:** Burke A. Cunha

**Affiliations:** * Winthrop-University Hospital, Mineola, New York, USA;; † SUNY School of Medicine, Stony Brook, New York, USA

**Keywords:** Alexander the Great, letter, West Nile virus

**To the Editor:** Marr and Calisher suggest the cause of Alexander the Great's death in Babylon in 323 B.C. was West Nile encephalitis (1). They were intrigued by the fact that as Alexander entered Babylon, ravens fell dead from the sky. The authors postulated the ravens might have had West Nile encephalitis, and because of the endemicity of mosquitoes in ancient Babylon, Alexander could have died of West Nile encephalitis. The authors are to be complimented on coming up with a novel explanation for his death, but this explanation has several problems (2,3).

Determining the exact cause of Alexander's death is impossible. Classical scholars are hampered by difficulties with translations from ancient Greek texts as well as differences in terms used by Plutarch in his description of Alexander's demise. We are left with a description that is incomplete, but nevertheless contains cardinal features of his terminal illness (4–6). In infectious disease practice, a syndromic diagnosis is the basis of the clinical approach. Astute infectious disease clinicians must discern between consistent and characteristic features in syndromic diagnosis. In addition to characteristic clinical features, syndromic diagnosis also depends on time relationships of clinical features. That splenomegaly is a feature of Epstein-Barr virus infectious mononucleosis is important, but equally as important is the late rather than early appearance of splenomegaly in the illness. A laundry list of features associated with various infectious diseases tells only part of the story and is diagnostically unhelpful unless placed in the proper time sequence.

In the authors' table, the clinical symptoms associated with Alexander's final days are listed (1). In my review of translations of ancient sources, chills are never mentioned as accompanying Alexander's slowly rising fever. After a steadily increasing fever, Alexander first became weak, then lethargic, and finally died after a 2-week febrile illness. These features and time course are inconsistent with various explanations that have been given for Alexander's death, i.e., influenza, poliomyelitis, alcoholic liver disease, malaria, schistosomiasis, leptospirosis, and poisoning (6–8).

The death of Alexander was certainly caused by an infectious disease and not poisoning or alcoholic liver disease. Although Alexander had an appetite for alcohol, his terminal illness is inconsistent with liver failure attributable to alcoholic cirrhosis or delirium tremens. Poisoning, which has been postulated by some, is not a reasonable diagnostic possibility either, since toxins or poisons are not accompanied by fever. Therefore, we are left with an infectious disease that was endemic in ancient Babylon and was fatal after approximately 2 weeks. The infectious disease that resulted in Alexander's demise was characterized by a slow but relentless increase in temperature during 2 weeks, unaccompanied by chills or drenching sweats. While remaining mentally alert, he drifted into an apathetic state, according to Alexander's Royal Diaries. Details of his death do not provide additional details other than he was febrile, weak, and gradually became lethargic, lapsed into coma, and died. Are the features of his illness and temporal sequence of events characteristic of West Nile encephalitis (9)?

West Nile encephalitis is a mosquito-borne infectious disease that may have been endemic in ancient Babylon. Ravens could have had West Nile encephalitis, and if West Nile encephalitis was present at the time, certainly it was transmitted to animals as well as humans. No one would argue with the possibility of West Nile encephalitis in the ancient Middle East; however, proving that West Nile encephalitis explains Alexander's death is more difficult. West Nile encephalitis begins acutely, with initial signs and symptoms of mental confusion and muscle weakness. Fevers are not usually the most conspicuous feature of West Nile encephalitis, and in most cases the fever does not usually increase or last more than a 2-week period. Other forms of viral encephalitis, including West Nile encephalitis, all begin with an abrupt change in mental status, e.g., encephalitis, at the outset of the illness. The patient's mental status may change over time, but encephalitic symptoms are present initially. This symptom is a characteristic feature of viral encephalitis, whether it is due to West Nile encephalitis or western equine encephalitis, Venezuelan equine encephalitis, St. Louis encephalitis, or Japanese encephalitis. Even non–arthropod-borne causes of viral encephalitis, e.g., herpes simplex virus I encephalitis, occurs with encephalitis as an initial, not terminal feature.

Alexander's final illness is more characteristic of typhoid fever than West Nile encephalitis. On Alexander's return to Babylon, he was confronted by many portents and omens and correctly assumed that they were a forewarning of his death. Not only were ravens falling from the sky, but the birds that were sacrificed to foretell the future were devoid of a liver lobe, which was thought by the ancients to be an ominous sign. A docile animal in the royal menagerie, in a violent outburst, kicked the royal lion to death. A mysterious person entered the royal chamber and sat on Alexander's throne bypassing the household guards. He claimed that he was divinely sent. West Nile encephalitis could explain these unusual phenomena.

However, the time course and characteristic clinical features of West Nile encephalitis are inconsistent with the cause of Alexander the Great's death (10). On the basis of characteristic features and time course of the illness, typhoid fever is the most likely explanation for Alexander the Great's death. The ravens in this case were the red herrings.
