# Supplementary material for: Alexander the Great and West Nile Virus Encephalitis
Source: Emerg Infect Dis. 2004 Jul;10(7):1328–33. doi: 10.3201/eid1007.040039 (PMC3323347; doi:10.3201/eid1007.040039)
Supplement: Related texts

# Reply: Alexander the Great and West Nile Virus Encephalitis

**DOI:** 10.3201/eid1007.040104

**Authors:** David Oldach, R. Michael Benitez, Philip A. Mackowiak

**Affiliations:** * VA Maryland Health Care System, Baltimore, Maryland, USA;; † University of Maryland School of Medicine, Baltimore, Maryland, USA

**To the Editor:** We wish to commend Marr and Calisher for their brilliant presentation of the West Nile virus (WNV) hypothesis to explain the death of Alexander the Great (1). Having recently proposed typhoid fever as the cause of Alexander's demise (2), we read their paper with particular interest. While we could argue the finer points of the WNV and typhoid hypotheses in explaining limited available clinical data, or perhaps debate the capacity of encephalitic ravens to perform the aerial acrobatics described by Plutarch, many of these considerations were thoughtfully anticipated by the authors. Instead, we have taken the opportunity to "Brush Up Our Plutarch." Reading widely through his essays, we have come to fear that Marr and Calisher, perhaps unaware of the magnitude of Plutarch's obsession with avian augeries, have been led down a feathered path. In story, after story, after story, birds portend.

Our source material was the Dryden translation, Volumes I and II, of Plutarch's Lives (3). We were immediately struck by the opening paragraph of his essay on Alexander, where he writes, "my design is not to write histories," and "I must be allowed to give my more particular attention to the marks and indications of the souls of men" (4). And so, the great writer served notice; particular details, especially where the material might lend insight into a man's character, were subject to a creative process that he himself could not describe as "history."

When approaching the time of Caesar's assassination, Plutarch wrote, "…many strange prodigies and apparitions are said to have been observed shortly before this event… the wild birds which perched in the forum" (5). As Cicero fled Antony's death sentence, Plutarch wrote, "…a flight of crows rose with a great noise, and made towards Cicero's vessel, as it rowed to land, and lighting on both sides of the yard, some croaked, others pecked the ends of the ropes" (6). On the founding of Rome, he wrote, "...concluding at last to decide the contest by a divination from a flight of birds… Remus, they say, saw six vultures, and Romulus double that number… Hence it is that the Romans, in their divinations from birds, chiefly regard the vulture" (7). (For Remus, who died shortly thereafter, this appears to have been a less propitious sighting.)

When writing on the lost grave of Theseus, Plutarch wrote, "…he, by chance, spied an eagle upon a rising ground pecking with her beak and tearing up the earth with her talons" (8).

On the defeat of the Persian armada at Salamis, he wrote, "...an owl was seen flying to the right hand of the fleet, which came and sat upon the top of the mast" (9). These examples, to which we could add others, should suffice to make our point.

Yet, we do not seek to diminish the contribution of Marr and Calisher. Plutarch, renown for his expositions on notable men, sought in doing so to identify elements of greatness. In this vein, we note the qualities that these three fine writers share. Truly, all are erudite. All share a remarkable awareness of the importance of birds. For this, both as physicians and as birders, we applaud them. In this age of emerging infections, including WNV and avian influenza viruses, we ignore bird health at our peril. We thank the doctors for this reminder and have increased our vigilance. We recommend, however, a grain of salt with Plutarch.

## References

[1] Marr JS, Calisher CH. Alexander the Great and West Nile virus encephalitis. Emerg Infect Dis. 2003;9:1599–603.1472528510.3201/eid0912.030288PMC3034319

[2] Oldach DW, Edwards R, Borza E, Benitez RM. A mysterious death. N Engl J Med. 1998;388:1764–9. 10.1056/NEJM1998061133824119625631

[3] Plutarch. Plutarch's lives. The Dryden translation. Originally published 1683–1686, with Revision in 1864. New York: The Modern Library, Random House, Inc.; 2001.

[4] Plutarch. Alexander. In: Plutarch's lives. The Dryden translation. Originally published 1683–1686, with Revision in 1864. Volume II. New York: The Modern Library, Random House, Inc.; 2001 p. 139.

[5] Plutarch. Caesar. In: Plutarch's lives. The Dryden translation. Originally published 1683–1686, with Revision in 1864. Volume II. New York: The Modern Library, Random House, Inc.; 2001. p. 239.

[6] Plutarch. Cicero. In: Plutarch's lives. The Dryden translation. Originally published 1683–1686, with Revision in 1864. Volume II. New York: The Modern Library, Random House, Inc., NY.; 2001. p. 440.

[7] Plutarch. Romulus. In: Plutarch's lives. The Dryden translation. Originally published 1683–1686, with Revision in 1864. Volume I. New York: The Modern Library, Random House, Inc.; 2001. p. 31.

[8] Plutarch. Theseus. In: Plutarch's lives. The Dryden translation. Originally published 1683–1686, with Revision in 1864. Volume I. New York: The Modern Library, Random House, Inc.; 2001. p. 24.

[9] Plutarch. Themistocles. In: Plutarch's lives. The Dryden translation. Originally published 1683–1686, with Revision in 1864. Volume I. New York: The Modern Library, Random House, Inc.; 2001. p. 155.

# Reply: Alexander the Great and West Nile Virus Encephalitis

**DOI:** 10.3201/eid1007.040396

**Authors:** Massimo Galli, Flavia Bernini, Gianguglielmo Zehender

**Affiliations:** * Istituto di Malattie Infettive e Tropicali, Università degli Studi di Milano, Milan, Italy

**To the Editor:** The article by Marr and Calisher (1) concerning the causes of the death of Alexander the Great triggered our curiosity about the possibility of supporting this hypothesis by determining the evolutionary time of West Nile virus (WNV). WNV is a member of the *Culex*-transmitted clade of flavivirus (which also includes Japanese encephalitis virus, St. Louis encephalitis virus, and Murray Valley encephalitis virus) whose reservoir is birds (1). Like most of the RNA viruses, flaviviruses are characterized by a high degree of genomic variability (2,3). Strains of WNV currently are divided into two distinct lineages on a molecular basis: one with a worldwide distribution and the other, which includes the prototypic strain isolated in Uganda in 1937 that is only found in sub-Saharan Africa and Madagascar.

To estimate the time of divergence among the different WNV strains, we conducted a phylogenetic analysis of a number of WNV sequences available in GenBank using a maximum likelihood (ML) method that makes it possible to estimate the branch lengths of a phylogeny with dated isolates under the SRDT (single rate dated tip) model (4). In particular, we retrieved sequences included in the envelope (E) gene of 38 WNV isolates: 18 lineage 1 strains representative of all of the proposed type 1 subtypes, including one Kunjin virus isolate (5), and 20 lineage 2 strains, including the original 1937 isolate from Uganda (6,7). The date of isolation was available for all of the viruses for which sequences where considered.

The 227-bp sequences were aligned with ClustalW (Thompson 1994), and distance-based unweighted pair group method with arithmatic mean (UPGMA) and ML methods were used to make the analysis. The distance matrix and the ML trees were obtained using the PAUP* program (version 4.0b10, Swafford 2001). The Kimura’s two-parameters model of nucleotide substitution was used with γ-distributed rates. The substitution model, α shape, Ti/Tv ratio and base frequencies were estimated using Modeltest version 3.06 (8). The trees were obtained by means of a DR heuristic search and were rooted by using Japanese encephalitis virus as the outgroup. The trees were used to estimate branch lengths in accordance with the single rate dated tips (SRDT) model using the Tipdate program implemented in PAML version 3.13 (9). A likelihood ratio test (LRT) was used to examine the fit of each model to the data. The high mean divergence between the two lineages (0.891 [SE 0.294] substitutions/site) was a good reason for analyzing them separately. The mean distance between the lineage 2 strains was 8.3 times shorter than that between the lineage 1 strains (0.018 [SE 0.05] sub/site vs. 0.154 [SE 0.036] sub/site). Analysis of the goodness-of-fit of the models showed that the likelihood of the SRDT and DR models was similar for lineage 2 (2 Δ lnL 26.04, degrees of freedom-df: 17-p > 0.05 LRT), whereas DR was significantly better than SRDT for lineage 1 (2 Δ lnL = 47.08, df = 15-p < 0.001 LRT).

The substitution rates estimated with the SRDT model were very similar in the two lineages (1.25 x 10^–4^ [±7.07 x 10^–6^] in lineage 1, and 1.20 x 10^–4^ [±7.03 x 10^–5^] in lineage 2). On the basis of these substitution rates, the most recent common ancestor (MRCA) for lineage 1 can be dated back 1,159 years ago (95% confidence interval [CI] 1,043–1,274, i.e., between 729 and 961 AD) and the MRCA for lineage 2 back to 208 years ago (95% CI 105–311; i.e., between 1,693 and 1,899 AD) (Figure).

**Figure:** Maximum likelihood (ML) phylogenies constructed under SRDT model for lineages 1 and 2 of West Nile virus. Horizontal branch lengths are proportional to time.

Our calculated substitution rates are very close to those reported for other RNA viruses, including some flaviviruses. A phylogenetic study of the entire E gene of various flaviviruses (3) estimated a rate of 7.5 x 10^–5^ nonsynonymous nucleotide substitutions/site/year, and the divergence times estimated on this basis showed that the *Flavivirus* genus is relatively young (<10,000 years). As suggested by the phylogenetic trees, the divergence of the three groups of *Flavivirus* (mosquito-borne, tick-borne, and no known vector viruses) is the earliest event in their evolution and dates back to no more than 5,000 years ago (2), and the divergence of the *Culex*-transmitted group (including WNV) and *Aedes*-transmitted flaviviruses (including dengue and yellow fever viruses) has been placed at approximately 3,200 years ago (3).

One possible limitation of our study is the fact that the goodness-of-fit of the DR model is better than that of the SRDT model for lineage 1. However, on the basis of the results of a simulation study, the estimated substitution rates should still be reliable indicators of the average rate of evolution and can be used to infer the divergence times correctly also in this case (10).

In conclusion, our divergence time estimate suggests that WNV is a relatively young virus and reduces the probability of incidental infections of humans before 1,000 years ago. Encephalitis itself became a frequent complication of WNV fever in 1996 (11), which suggests the recent appearance of more pathogenic viral strains. Although the present spread of WNV lineage 1 may be compatible with its presence in the geographic area of ancient Babylon, the molecular dating of its origin acquits it of any responsibility for Alexander’s death.

## References

[1] Marr JS, Claisher CH. Alexander the Great and West Nile virus encephalitis. Emerg Infect Dis. 2003;9:1599–603.1472528510.3201/eid0912.030288PMC3034319

[2] Gaunt MW, Sall AA, de Lamballerie X, Falconar AK, Dzhivanian TI, Gould EA. Phylogenetic relationships of flaviviruses correlate with their epidemiology, disease association and biogeography. J Gen Virol. 2001;82:1867–76.1145799210.1099/0022-1317-82-8-1867

[3] Zanotto PM, Gould EA, Gao GF, Harvey PH, Holmes EC. Population dynamics of flaviviruses revealed by molecular phylogenies. Proc Natl Acad Sci U S A. 1996;93:548–53. 10.1073/pnas.93.2.5488570593PMC40088

[4] Rambaut A. Estimating the rate of molecular evolution: incorporating non-contemporaneous sequences into maximum likelihood phylogenies. Bioinformatics. 2000;16:395–9. 10.1093/bioinformatics/16.4.39510869038

[5] Lanciotti RS, Gubler DJ, Trent DW. Molecular evolution and phylogeny of dengue-4 viruses. J Gen Virol. 1997;78:2279–84.929201510.1099/0022-1317-78-9-2279

[6] Smithburn KC, Hughes TP, Burke AW, Paul JA. Neurotropic virus isolated from the blood of a native of Uganda. Am J Trop Med. 1940;20:471–92.

[7] Burt FJ, Grobbelaar AA, Leman PA, Anthony FS, Gibson GV, Swanepoel R. Phylogenetic relationships of southern African West Nile virus isolates. Emerg Infect Dis. 2002;8:820–6.1214196810.3201/eid0808.020027PMC2732512

[8] Posada D, Crandall KA. MODELTEST: testing the model of DNA substitution. Bioinformatics. 1998;14:817–8. 10.1093/bioinformatics/14.9.8179918953

[9] Yang Z. PAML: a program package for phylogenetic analysis by maximum likelihood. CABIOS. 1997;13:555–6.936712910.1093/bioinformatics/13.5.555

[10] Jenkins GM, Rambaut A, Pybus OG, Holmes EC. Rates of molecular evolution in RNA viruses: a quantitative phylogenetic analysis. J Mol Evol. 2002;54:156–65. 10.1007/s00239-001-0064-311821909

[11] Tsai TF, Popovici F, Cernescu C, Campbell GL, Nedelcu NI. West Nile encephalitis epidemic in southeastern Romania. Lancet. 1998;352:767–71. 10.1016/S0140-6736(98)03538-79737281

# Reply: Alexander the Great and West Nile Virus Encephalitis

**DOI:** 10.3201/eid1007.040320

**Authors:** John S. Marr, Charles H. Calisher

**Affiliations:** * Virginia Health Department, Richmond, Virginia, USA;; † Colorado State University, Fort Collins, Colorado, USA

**In reply:** The response by Oldach et al. is wonderfully whimsical (a word that was unfortunately deleted from our manuscript by the EID reviewers). We do not think a witty response is indicated and cannot think of one anyway. We note, however, that both our paper (1) and the Oldach et al. response are examples of the serendipitous pleasures that can be brought to the literature by classical citations, and that all orthodox medical theories on historical causations should be periodically reexamined. Because we are the type who do not mind crawling around in dirty places, we consider computer-based epidemiology for the birds and are willing to eat *Corvus brachyrhynchos*.

We also thank Cunha for his exhaustive differential diagnosis. We felt we had to address all previously cited diagnoses as well as those not posited in the literature, even though, like Cunha, we did not think most of them were likely causes. We concur that most plant toxins do not induce fever, but some do contain anticholinergic alkaloids that may interfere with perspiration and elevate body temperature. (A most enjoyable recent book discusses a variety of poisons and their widespread use by the Greeks, Romans and Scythians [2]. The book illuminates the widespread use of poisons not only on persons but also as weapons in battle and sieges.) Since thermometers were not available at that time, it remains impossible to document this critical vital sign, but since poisoning was specifically mentioned by Plutarch, we felt we could not ignore this possibility. Who are we to ignore Plutarch?

We also agree that typhoid fever remains high on the list of probable causes, as Oldach eloquently argued 5 years ago (3). Although individual cases of this disease usually occur in a camp setting, one would expect reports of other similar cases (the same for malaria), which was apparently not the case. A singular case of West Nile encephalitis, however, is the rule, not the exception.

Cunha stresses the importance of "acute infectious diseases clinicians" arriving at a procrustean diagnosis. In our diagnosis, we chose to emphasize previously overlooked environmental and public health considerations, such as climatic conditions and the deaths of ravens. As stated earlier, we also had an ulterior motive in our writing: to continue the legacy of others in heuristic discussions of the classics (4). In that sense, we have achieved our goal. Cunha considers the diagnosis of West Nile encephalitis as a "red herring." We point out that *Clupeus harengus* was quite bountiful in ancient times (5), and at least some must have been erythematous.

As for the marvelous letter from Galli, Bernini, and Zehender, which minimizes Plutarch's assertions, we can only say that perspective is everything. That these investigators have gone to such lengths to investigate our "best guess" is reward enough for us. We attempted to show retrospectively, as all diagnoses must be done for dead patients, that to come to an Occamic conclusion, one should at least have a look beyond the obvious. Given the multitude of letters and messages we have received since the publication of our article, and given all the interviews we have given to newspapers, magazines, and other media, which always prefer a "hot" topic to an important one, we have been successful in promoting intellectual debate. We would be delighted to be proven right or wrong in our thesis, but we are not convinced that Galli et al. are correct in their estimation that West Nile virus did not exist at the time of the death of Alexander the Great. Various phylogenetic studies of flaviviruses (6–8) have discussed the time period when flaviviruses have emerged or diverged, with estimates based on nucleotide substitution rates. However, most groups seem to be retreating from their former definitive positions on this subject because of various technical discrepancies originating from assumptions made regarding the sequence dating methods themselves. Some investigators believe that such dating methods are unreliable for all but the most recent divergence events. At the very least, these methods remain controversial, as does the cause of death of Alexander the Great, who is, after all these years, still causing trouble.

## References

[1] Marr JS, Calisher CH. Alexander the Great and West Nile virus encephalitis. Emerg Infect Dis. 2003;9:1599–603.1472528510.3201/eid0912.030288PMC3034319

[2] Mayor A. Greek fire, poison arrows and scorpion bombs: biological and chemical warfare in the Ancient World. New York: Overlook Press; 2003.

[3] Oldach DW, Richard RE, Borza EN, Benitz RM. A mysterious death. N Engl J Med. 1998;338:1764–9. 10.1056/NEJM1998061133824119625631

[4] Langmuir AD, Ray CG. The Thucydides syndrome. JAMA. 1987;257:3071. 10.1001/jama.1987.033902200690223295311

[5] Kriiska A, Lõugas L, Saluäär U. Archeological excavations of the stone age settlement site and ruin of the stone cist grave of the early metal age in Kasekula. Available from: http://ethesis.helsinki.fi/julkaisut/hum/kultt/vk/kriiska/tekstid/09.html

[6] Zanotto PM, Gould EA, Gao GF, Harvey PH, Holmes EC. Population dynamics of flaviviruses revealed by molecular phylogenies. Proc Natl Acad Sci U S A. 1996;93:548–53. 10.1073/pnas.93.2.5488570593PMC40088

[7] McGuire K, Holmes EC, Gao GF, Reid HW, Gould EA. Tracing the origins of louping ill virus by molecular phylogenetic analysis. J Gen Virol. 1998;79:981–8.960331210.1099/0022-1317-79-5-981

[8] Gould EA, de Lamballerie X, Zanotto PM, Holmes EC. Evolution, epidemiology, and dispersal of flaviviruses revealed by molecular phylogenies. Adv Virus Res. 2001;57:71–103. 10.1016/S0065-3527(01)57001-311680389
